# The Simpson grading: defining the optimal threshold for gross total resection in meningioma surgery

**DOI:** 10.1007/s10143-020-01369-1

**Published:** 2020-08-18

**Authors:** Benjamin Brokinkel, Dorothee Cäcilia Spille, Caroline Brokinkel, Katharina Hess, Werner Paulus, Eike Bormann, Walter Stummer

**Affiliations:** 1 Department of Neurosurgery, University Hospital Münster, Albert-Schweitzer-Campus 1, Building A1, 48149 Munster, Germany; 2 Institute for Clinical Radiology, University Hospital Münster, Münster, Germany; 3 Institute of Neuropathology, University Hospital Münster, Münster, Germany; 4 Institute of Biostatistics and Clinical Research, University Münster, Münster, Germany

**Keywords:** Meningiomas, Microsurgery, Progression, Recurrence, Simpson grading

## Abstract

Classification of the extent of resection into gross and subtotal resection (GTR and STR) after meningioma surgery is derived from the Simpson grading. Although utilized to indicate adjuvant treatment or study inclusion, conflicting definitions of STR in terms of designation of Simpson grade III resections exist. Correlations of Simpson grading and dichotomized scales (Simpson grades I–II vs ≥ III and grade I–III vs ≥ IV) with postoperative recurrence/progression were compared using Cox regression models. Predictive values were further compared by time-dependent receiver operating curve (tdROC) analyses. In 939 patients (28% males, 72% females) harboring WHO grade I (88%) and II/III (12%) meningiomas, Simpson grade I, II, III, IV, and V resections were achieved in 29%, 48%, 11%, 11%, and < .5%, respectively. Recurrence/progression was observed in 112 individuals (12%) and correlated with Simpson grading (*p* = .003). The risk of recurrence/progression was increased after STR in both dichotomized scales but higher when subsuming Simpson grade ≥ IV than grade ≥ III resections (HR: 2.49, 95%CI 1.50–4.12; *p* < .001 vs HR: 1.67, 95%CI 1.12–2.50; *p* = .012). tdROC analyses showed moderate predictive values for the Simpson grading and significantly (*p* < .05) lower values for both dichotomized scales. AUC values differed less between the Simpson grading and the dichotomization into grade I–III vs ≥ IV than grade I–II vs ≥ III resections. Dichotomization of the extent of resection is associated with a loss of the prognostic value. The value for the prediction of progression/recurrence is higher when dichotomizing into Simpson grade I–III vs ≥ IV than into grade I–II vs ≥ III resections.

## Introduction

Microsurgical resection remains the treatment of choice for most symptomatic and/or space-occupying meningiomas [5]. In 1957, D. Simpson described a simple method for intraoperative assessment of the extent of tumor removal and further showed correlations with the risk of postoperative recurrence [20]. Nowadays, the Simpson classification system is widely used for semiquantitative assessment of the extent of resection in meningioma surgery and established in both clinical routine and research [5].

Along with numerous reports about correlations between the Simpson grading and the risk of postoperative tumor relapse, derived dichotomous scales distinguishing gross and subtotal resection (GTR and STR) have been introduced and are, nowadays, commonly used to quantify the extent of tumor removal in both retrospective [2–4, 6–8, 13, 14, 22, 23] but also currently ongoing prospective clinical trials [10, 17]. Remarkably, the definitions of both dichotomizations, particularly with regard to the classification of Simpson grade III resections, remain controversial. In fact, contradictive descriptions are even found when comparing current meningioma treatment guidelines with the pioneering work from D. Simpson [5, 20]. Hence, while some might argue that the disposition of Simpson grade III surgeries as GTR or STR is basically academic, the designation potentially impacts decision making towards adjuvant treatment (e.g., in high-grade meningiomas [5]) and study inclusion. Moreover, a uniform designation would be helpful for the interpretation of previous studies and should be particularly strived for in future clinical trials.

Hypothesizing relevant differences in their prognostic values, we here present comparative analyses of the utility of both commonly reported ways of dichotomization of the extent of resection for the prediction of postoperative tumor progression using multivariate Cox regression models and time-dependent receiver operating characteristic (ROC) analyses in a large volume series.

## Materials and methods

### Data recovery

Data were recovered from the local meningioma data base and have been extensively described previously [1, 2, 15, 19, 23]. Briefly, archives of the Institute of Neuropathology were reviewed for all histopathologically confirmed meningiomas resected in our department between 1991 and 2018. Neuropathological diagnosis and histopathological grading had been performed according to the current 2016 WHO classification in all cases [16]. Clinical and radiological data included age at diagnosis, sex, preoperative Karnofsky Performance Score (KPS), and the tumor location, classified into “skull base” and “non-skull base” position. Surgery had been indicated for progressive lesions inaccessible for radiosurgical treatment and for symptomatic and/ or space occupying tumors. Maximum safely achievable tumor resection or reduction was performed in all patients, and the extent of resection was classified intraoperatively according to the Simpson classification by the attending neurosurgeon, as it is standard in our institution. As we aimed to analyze the prognostic value of the intraoperatively assessed extent of resection and, furthermore, postoperative imaging was not considered to further classify the extent of resection. Adjuvant irradiation was recommended for primary diagnosed anaplastic and recurrent or subtotally resected atypical meningiomas as well as for benign lesions after debulking. None of the patients received chemotherapy for meningioma treatment. Patients were followed up by clinical examinations and magnetic resonance imaging (MRI). In detail, contrast-enhanced MRI was performed 3 months after surgery and then repeated in 12- and 6-month intervals in grade I and high-grade meningiomas, respectively. Tumor progression was evaluated by a team of two independent observers, including at least one neurosurgeon and one (neuro-)radiologist. Progression was diagnosed in cases of any detected tumor growth. Contrast-enhanced CT scans were performed in patients with contraindications against MRI. Data about progression were additionally updated by standardized questionnaires, which were sent to the primary care takers. Data collection and scientific use were approved by the local ethics committee (Münster 2018-061-f-S).

### Statistical analyses

Data are described by standard statistics using standard commercial statistic software (IBM SPSS Statistics, Version 24, IBM, Germany, SAS version 9.4, SAS Institute, North Carolina, USA and R Version 3.6.2). Continuous variables are described by median and range and compared using Mann–Whitney *U* test. Categorical variables are described by absolute and relative frequencies and compared using Fishers exact. Progression free interval (PFI) was defined as the duration between the date of surgery and the date of progression or, in case of an event free survival, until the date of last follow-up. PFI was estimated by Kaplan–Meier analyses and compared by log-rank tests. Univariate and multivariate analyses for tumor progression were performed using backward Wald logistic regression and characterized by hazard ratios (HR), 95% confidence intervals (CI), and Wald-test *p* values. The following variables were included in the multivariate analysis using Cox proportional hazard models: age, sex (female (ref) vs. male), WHO-grade (classified into grade I (ref) vs. II/III (high-grade) histology), tumor location (dichotomously classified as skull base (ref) vs non-skull base), and degree of resection, classified as described in the corresponding text sections. Ability to predict postoperative tumor progression was further compared by AUC (area under the curve) values in time-dependent ROC analyses, using the R-package “timeRoc” [11]. A *p* < .05 was considered to be statistically significant throughout the entire analyses. All reported *p* values are two-sided.

## Results

**Fig. 1:**
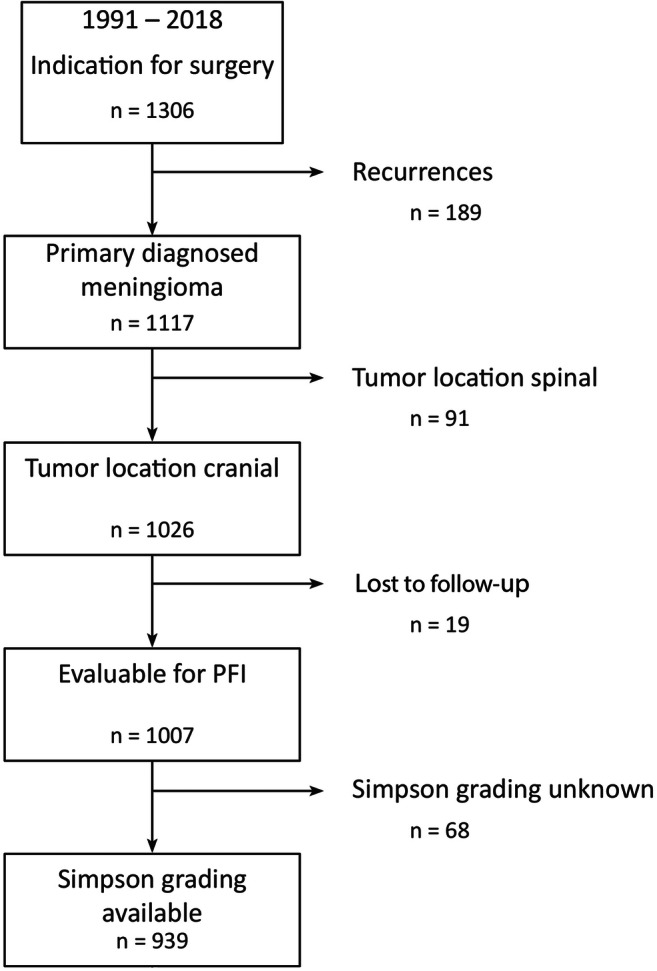
Flowchart of patient selection. After exclusion of patients with recurrent or spinal lesions and with missing data about outpatient follow-up and the extent of resection, 939 cases were subjected to analyses

Using the above described approach (Fig. 1), 939 patients including 268 (28%) males and 671 (72%) females (median age 58 years, range 7–91) with primary diagnosed intracranial meningioma were identified and subjected to further statistical analyses. Tumors were located at the convexity in 328 (35%), falcine/parasagittal in 126 (13%), at the skull base in 419 (45%), in the posterior fossa in 55 (6%), and intraventricular in 11 cases (1%). Simpson grade I, II, III, IV, and V resections were achieved in 280 (29%), 446 (48%), 103 (11%), 106 (11%), and 4 (< .5%) cases, respectively. Correspondingly, 726 patients (77%) with Simpson grade I–II resections were assigned to the GTR-1 group, and 213 individuals (23%) to the STR-1 group. Applying the second way of dichotomization, 829 patients (88%) with Simpson grade I–III resections were allotted to the GTR-2, and 110 cases (12%) to the STR-2 group. Neuropathological analyses revealed WHO grade I and grade II/III histology in 825 (88%) and 114 (12%) patients, respectively. Preoperative Karnofsky Performance Score was available in 936 patients (99%) and was ≥ 80 in 805 cases (86%). In 711 patients with available data (75%), adjuvant irradiation was administered in 30 cases (4%).

### The Simpson grading correlates with the risk of postoperative progression

**Fig. 2:**
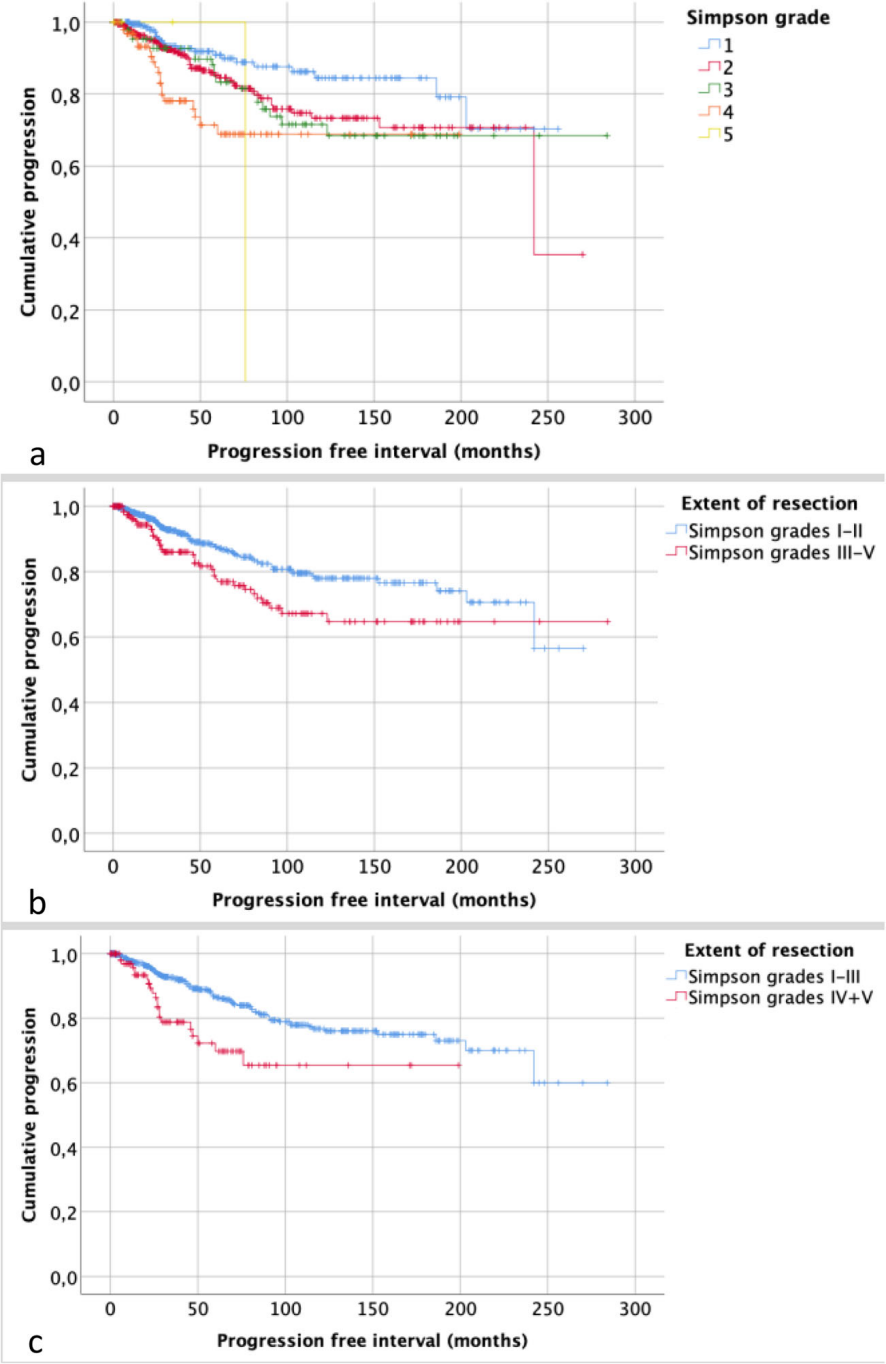
Kaplan–Meier plots showing correlations between the extent of resection and progression. PFI correlated with the Simpson grade (*p* = .003, **a**) and was also shorter after STR as compared with GTR after dichotomization into Simpson I-II vs ≥ III (*p* = .007, **b**) and Simpson I-III vs ≥ IV (*p* = .001, **c**)

Within a median follow-up period of 37 months (range: 0–284 months), tumor recurrence or progression was observed in 112 individuals (12%). Table 1 summarizes correlations of clinical and histopathological data with recurrence. Recurrence or progression was observed in 21 (8%), 51 (11%), 19 (18%), 20 (19%), and 1 patients (25%) following Simpson grade I, II, III, IV, and V resections (*p* = .003), respectively, and PFI correlated with the Simpson grading (*p* = .003; Fig. 2). Dichotomous analyses further revealed a higher risk of progression after Simpson grade III than after Simpson grade II resections (HR: 1.56, 95%CI 1.01–2.42; *p* = .045). Multivariate analyses adjusted for patients’ age, sex, tumor location, and high-grade histology confirmed an increased risk of progression after Simpson grade II (HR: 1.73, 95%CI 1.04–2.87; *p* = .035) and IV resections (HR: 3.23, 95%CI 1.74–6.00; *p* < .001) with a similar trend following Simpson grade III surgery (HR: 1.85, 95%CI .99–3.43; *p* = .053). In contrast, Simpson grade V resections were not correlated with progression (HR: 5.70, 95%CI .76–42.51; *p* = .090).

**Table 1:** Correlations between clinical and histopathological variables and progression. Male gender, the extent of resection, and high-grade histology were found to correlate with prognosis in both univariate (left column) and multivariate (right column) analyses. To avoid collinearity, Simpson grade and the dichotomized extent of resection were not put into the multivariate model at the same time. Of the latter, results from multivariate analyses are given in the manuscript text

Variable	HR^1^, 95%CI^2^	*p* value^3^	HR, 95%CI	*p* value^4^
Age	1.01, .99–1.02	.368	1.01, .99–1.02	.443
Sex				
Female (ref^5^) vs male	2.24, 1.54–3.24	< .001	1.63, 1.10–2.40	.015
Tumor location				
Non-skull base (ref) vs skull base	1.31, .90–1.89	.158	1.23, .83–1.83	.303
WHO grade				
Grade I (ref) vs high-grade histology	4.44, 3.03–6.50	< .001	4.46, 2.96–6.72	<.001
Simpson grade				
I	ref		ref	
II	1.73, 1.04–2.87	.035	1.74, 1.04–2.94	.036
III	1.85, .99–3.43	.053	1.76, .93–3.33	.080
IV	3.23, 1.74–6.0	< .001	3.86, 2.01–7.42	< .001
V	5.70, .76–42.51	.090	3.35, .44–25.64	.245
Dichotomized scales				
Simpson grade I/II (ref) vs ≥ III	1.68, 1.14–2.48	.008	n/a^6^	
Simpson grade I–III (ref) vs ≥ IV	2.20, 1.36–3.56	.001	n/a	

*n/a* not applicable

^1^Hazard ratio

^2^Confidence interval

^3^Univariate backward Wald *p* value

^4^Multivariate backward Wald *p* value

^5^Reference

### Subtotal resection according to both dichotomizations predicts progression

**Fig. 3:**
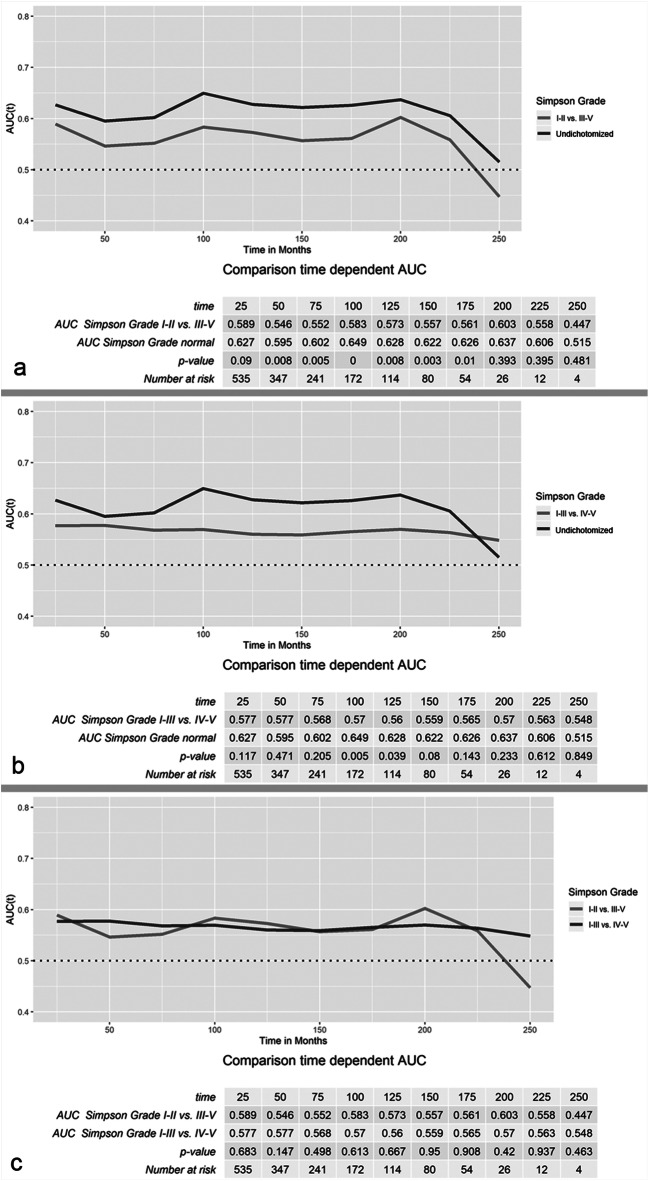
Time-dependent ROC analyses of the predictive value of different systems of classification of the extent of resection. In **a**, the course of the AUC values of the dichotomized extent of resection (Simpson grade I–II vs ≥ III) and the undichotomized Simpson grading runs almost in parallel. However, AUC values of the dichotomized scale are significantly lower up to 175 months (*p* < .05). In contrast, AUV values after dichotomization into Simpson grade I–III vs ≥ IV resections differed less significantly (**b**). However, in direct comparison, AUC values of both dichotomization scales did not significantly differ during the entire observation period

After dichotomization, progression was observed in 72 patients after GTR-1 and 40 cases after STR-1 (10 vs 19%, *p* = .001). On the other hand, progression was found after GTR-2 and STR-2 in 91 and 21 individuals, respectively (11 vs 19%, *p* = .018). Similarly, PFI was significantly shorter after STR in both dichotomization groups (Fig. 3). Correspondingly, STR after both ways of dichotomizations was associated with an increased risk of tumor relapse (*p* < .05; Table 1). In multivariate analyses, STR in terms of Simpson grade ≥ III (HR: 1.67, 95%CI 1.12–2.50; *p* = .012) and, even more, Simpson grade ≥ IV (HR: 2.49, 95%CI 1.50–4.12; *p* < .001) was strongly correlated with progression.

### Comparative analyses of the predictive value of different systems of quantification of the extent of resection

Finally, time-dependent ROC analyses were performed to compare the value of both ways of dichotomization of the extent of resection and the Simpson grading for the prediction of tumor recurrence/progression (Fig. 3). Remarkably, AUC values over the entire observation period were found to be low to moderate in all three analyzed quantification scales (range: .447–.649). On the one hand, the ROC curve of the dichotomized extent of resection (Simpson grade I–II vs. ≥ III) ran in parallel to the undichotomized Simpson grading system. However, AUC values of the dichotomized extent of resection were found to be distinctly lower during most of the follow-up period (Fig. 3a). On the other hand, AUC values after dichotomization into Simpson grade I–III vs ≥ IV only differed between 100 and 125 months after surgery from the AUC values of the undichotomized Simpson grading system (Fig. 3b). In direct comparison, none of the analyzed dichotomized scales was significantly superior in the prediction of tumor progression over the entire observation period (Fig. 3c).

## Discussion

The Simpson classification system and derived dichotomized scales provide an easily applicable assessment of the extent of resection and important information about the risk of postoperative tumor recurrence. As potentially impacting both adjuvant treatment and study inclusion [5], a standardized terminology of GTR in terms of designation of Simpson grade III resections should be self-evident.

As expected, we found the Simpson grading to be significantly correlated with tumor recurrence in univariate and multivariate analyses. This observation matches findings from numerous previous studies, reporting the extent of resection as a strong predictor for tumor progression [3, 4, 6, 13, 14, 20]. The lack of correlation of Simpson grade V resection with progression in our study can be presumably explained by the low number of patients and events in this group (*N* = 1 of 4, 25%). Similarly, STR according to both ways of dichotomization was strongly correlated with progression. However, hazard ratios and corresponding *p*-values after both univariate and multivariate analyses suggested a higher predictive value of the dichotomization into Simpson grade I–III vs ≥ IV resections.

In addition to numerous previous studies reporting correlations between the Simpson grading and recurrence, we also provide direct comparative analyses of the value of the three quantification scales of the extent of resection for the prediction of tumor progression. Despite strong correlations with recurrence, the AUC values of the Simpson grading as well as both ways of dichotomizations ranged from .447 to .649, thus indicating only a moderate prognostic value. Remarkably, AUC values of both dichotomized scales during middle- and long-term follow-ups were found lower as compared with the Simpson grading system, reflecting a loss of predictive value by dichotomization. The AUC curves of the Simpson grading and after dichotomizing into Simpson grade I–II vs ≥ III resections basically ran in parallel, presumably caused by the similar cohorts after only subsuming two groups of Simpson grades as GTR during dichotomization. However, statistical analyses revealed significantly lower AUC values of the latter. On the other hand, although the course of the AUC curve of the Simpson grading and after dichotomizing into Simpson grade I–III vs ≥ IV resections differed on visual inspection, these differences were statistically less significant as compared with the Simpson grade I–II vs ≥ III dichotomization. However, in direct comparison, none of the AUC values of both dichotomized scales was found to be superior for the prediction of recurrence in statistical analyses.

Altogether, we found marginal differences of the prognostic value of both ways of dichotomizations of the extent of resections. The results from both Cox regression and time-dependent ROC analyses suggest that dichotomization into Simpson grade I–III vs grade ≥ IV resections allows a more exact prediction of the risk of postoperative tumor relapse than the classification into Simpson grade I–II vs ≥ III surgeries. Hence, irrespective of the discussion if bipolar coagulation of the dura attachment is more radical than simple dissection of the tumor from a biological point of view, these findings are in favor for a designation of Simpson grade I–III resections as GTR also for statistical reasons.

Although providing detailed statistical analyses in a large patient collective, the authors are aware of some limitations of the study. The Simpson grades were retrospectively obtained from operative reports and not adjusted after postoperative imaging, hence potentially suffering from inaccurate intraoperative assessment or bias in some cases. In fact, postoperative imaging in another series revealed an intraoperative overrating of the extent of resection in a considerable portion of surgeries [21]. As data were only gained from one tertiary neurosurgical department, a general transferability of our results remains unclear and should be further investigated in multicenter analyses. While histopathological grading was performed according to the current WHO classification of brain tumors in all cases, data about molecular alterations such as TERT mutations or DNA methylation were not available but have been shown to distinctly impact prognosis [12, 18]. Finally, although follow-up imaging was carefully analyzed, progression was diagnosed in case of any tumor growth but not according to previously proposed RANO criteria [9].

In conclusion, any dichotomization of the extent of resection was found to be associated with a reduction of prognostic value. Although marginal, the prognostic value for the prediction of progression was higher when dichotomizing into Simpson grade I–III vs ≥ IV than into Simpson grade I–II vs ≥ III resections, hence favoring a corresponding uniform definition of GTR and application during clinical trials and care for meningioma patients.

## Data Availability

None.
