# Correction to: The Simpson grading: defining the optimal threshold for gross total resection in meningioma surgery

**DOI:** 10.1007/s10143-021-01629-8

**Published:** 2021-09-21

**Authors:** Benjamin Brokinkel, Dorothee Cäcilia Spille, Caroline Brokinkel, Katharina Hess, Werner Paulus, Eike Bormann, Walter Stummer

**Affiliations:** 1 Department of Neurosurgery, University Hospital Münster, Albert-Schweitzer-Campus 1, Building A1, 48149 Munster, Germany; 2 Institute for Clinical Radiology, University Hospital Münster, Münster, Germany; 3 Institute of Neuropathology, University Hospital Münster, Münster, Germany; 4 Institute of Biostatistics and Clinical Research, University Münster, Münster, Germany

**Correction to: Neurosurgical Review (2021) 44:1713–1720**

10.1007/s10143-020-01369-1

The article “The Simpson grading: defining the optimal threshold for gross total resection in meningioma surgery”, written by Benjamin Brokinkel, Dorothee Cäcilia Spille, Caroline Brokinkel, Katharina Hess, Werner Paulus, Eike Bormann, and Walter Stummer, was originally published Online First without Open Access. After publication in volume 44, issue 3, page 1713–1720, the author decided to opt for Open Choice and to make the article an Open Access publication. Therefore, the copyright of the article has been changed to ©The Author(s) 2020 and the article is forthwith distributed under the terms of the Creative Commons Attribution 4.0 International License, which permits use, sharing, adaptation, distribution and reproduction in any medium or format, as long as you give appropriate credit to the original author(s) and the source, provide a link to the Creative Commons licence, and indicate if changes were made. The images or other third party material in this article are included in the article’s Creative Commons licence, unless indicated otherwise in a credit line to the material. If material is not included in the article’s Creative Commons licence and your intended use is not permitted by statutory regulation or exceeds the permitted use, you will need to obtain permission directly from the copyright holder. To view a copy of this licence, visit http://creativecommons.org/licenses/by/4.0/. Open Access funding enabled and organized by Projekt DEAL.

The original article has been corrected.

